# Promoting respectful maternity care for adolescents in Ghana: a quasi-experimental study protocol

**DOI:** 10.1186/s12978-020-00977-w

**Published:** 2020-08-24

**Authors:** Helen H. Habib, Kwasi Torpey, Ernest Tei Maya, Augustine Ankomah

**Affiliations:** 1 Department of Population, Family and Reproductive Health, School of Public Health, College of Health Sciences, University of Ghana, Accra, Ghana; 2 Population Council Ghana, Accra, Ghana

**Keywords:** Intra-partum mistreatment, Maternal mortality, Adolescent sexual reproductive health and rights, Ghana

## Abstract

**Background:**

Intra-partum mistreatment by healthcare providers remains a global public health and human rights challenge. Adolescents, who are typically younger, poorer and less educated have been found to be disproportionately exposed to intra-partum mistreatment. In Ghana, maternal mortality remains a leading cause of death among adolescent females, despite increasing patronage of skilled birth attendance in health facilities. In response to the the World Health Organisation Human Reproduction Programme (WHO-HRP) recommendations to address mistreatment with Respectful Maternity Care (RMC), this study aims to generate evidence on promoting respectful treatment of adolescents using an intervention that trains health providers on the concept of mistreatment, their professional roles in RMC and the rights of adolescents to RMC.

**Methods:**

This study will employ a pre-test post-test quasi-experimental design. At pre-test and post-test, quantitative surveys will be conducted among adolescents who deliver at health facilities about their labour experience with mistreatment and RMC. A total target of 392 participants will be recruited across intervention and control facilities. Qualitative interviews will also be conducted with selected adolescents and health professionals for an in-depth understanding of the phenomenon. Following the pre-test, a facility-based training module will be implemented at intervention facilities for the facility midwives. The modules will be co-facilitated by the principal investigator and key resource persons from the district health directorate Quality of Care teams. Training will cover the rights of adolescents to quality healthcare, classifications of mistreatment, RMC as a concept and the role of professionals in providing RMC. No intervention will occur in the control facilities. Descriptive statistics, logistic regressions and difference in differences analyses will be computed. Qualitative data will be transcribed and thematically analysed.

**Discussion:**

This study is designed to test the success of an intervention in promoting RMC and reducing intra-partum mistreatment towards adolescents. It is expected that the findings of this study will be beneficial in adding to the body of knowledge in improving maternal healthcare and reducing maternal mortality, especially for adolescents.

**Trial registration:**

Name of the registry: Pan African Clinical Trials Registry. PACTR202008781392078.

## Plain English summary

Mistreatment during childbirth remains a well-documented worldwide challenge, especially among poorer, younger and less educated women. In Ghana, the most vulnerable groups include adolescents, who also happen to bear a disproportionately high risk of maternal mortality. Despite the increasing rates of facility-based births which are attended to by trained obstetric professionals, maternal mortality persists as a public health challenge in Ghana and sub-Saharan Africa in general. Following landscape studies which suggest that mistreatment during delivery may be a strong predictor for poor birth outcomes, the WHO has recommended that this issue be addressed with respectful maternity care. This study, therefore, aims to determine the effectiveness of a training intervention for health providers targeted at reducing mistreatment of adolescents during childbirth. This will contribute to knowledge and inform programming and policy on improving maternal healthcare and reducing maternal mortality and morbidity, especially among adolescents.

## Background

Mistreatment suffered by women who deliver within health facilities is an extensively documented phenomenon [1–3]. The most reported forms of facility-based mistreatment which often occur in a continuum are categorized as physical abuse, non-consented care, non-confidential care, non-dignified care, discrimination based on specific patient attributes, abandonment of care, and detention in facilities [4]. Whilst intrapartum mistreatment traverses geographical boundaries and socio-economic levels, [1, 2, 5–7] certain factions of women appear to bear a markedly high burden of the phenomenon [8–10]. As a group of interest, adolescent women appear disproportionately predisposed to disrespectful treatment attributable to their young age, lower educational attainment, lower socio-economic status, lack of autonomy, as well as provider and social prejudices about their engagement in pre-marital and early sex [11–15]. Mistreatment in service provision for pregnant adolescents is problematic as it compounds the already existing burden of social and health risks [11, 16–18]. Currently, maternal mortality remains one of the leading global causes of death among adolescents, especially in sub-Saharan Africa and is considered a pressing public health challenge [19]. Approximately 11% of births worldwide occur among adolescent girls aged between 15 and 19 years old, majority of which occur in low to middle income countries [20]. In Ghana, this proportion of 14% is approximately a third higher than the global average [21, 22]. Despite the absence of nationally representative data on what proportion of maternal deaths are adolescents, research suggests adolescents may constitute a larger proportion of maternal mortality and morbidity figures since they bear a higher risk of pregnancy-related complications than their older counterparts [23]. A WHO Multi-country Survey on Maternal and Newborn Health revealed that adolescents 19 years and below were twice as likely to develop eclampsia than women over 20 years of age [24]. The same study reported a 50% higher probability of systemic infections among adolescents and a 13% higher likelihood of severe maternal outcomes as compared to women over the age of 20 years. Studies investigating efficacious strategies which may successfully alleviate this burden have identified appropriately-timed intrapartum interventions to be the most beneficial means of reducing maternal mortality and morbidity compared to antenatal and postpartum interventions [25–27]. These include promoting facility-based births and as a corollary, increasing skilled birth attendance to enable early identification and management of complications [27, 28]. Accordingly, interventions in Ghana have targeted overcoming barriers to facility access especially geographic and financial factors. Financial access has been improved by waiving antenatal care and delivery fees whilst the establishment of Community-Based Health Planning Service Compounds (CHPS-Compound) has improved provision of maternal care to the last mile within communities [29–31]. Nonetheless, the proportion of facility-based births in Ghana at 78% is currently less than the global average of 81% [22]. Additionally, the national maternal mortality rate of 308 per 1000 births is still markedly higher than the global average of 211 per 1000 births and far short of the SDG target of 70 per 1000 births [21]. Within the five years preceding the 2017 Ghana Maternal and Health Survey, only 71% of pregnant adolescents utilized facility-based deliveries [22]. This has been linked to the mistreatment parturients face during facility-based births [32, 33]. This data suggests that attempts to improve maternal mortality and morbidity may be hindered by the mistreatment of adolescents during childbirth.

Consequently, the Human Reproduction Programme of the World Health Organization (WHO-HRP), has prescribed recommendations on addressing mistreatment of women during childbirth with respectful maternity care [34]. Respectful Maternity Care (RMC) refers to the organization and management of health systems in a manner that ensures respect for women’s sexual and reproductive health and human rights [34]. Indeed, the absence of RMC has been identified as a key disincentive to accessing facility-based births; even in contexts where financial and physical access are not necessarily problematic [14, 32, 35]. Studies have also reported adverse clinical outcomes like prolonged labour, unnecessary pain, haemorrhage, mental distress and in extreme cases, death [36–39] during facility based births where RMC is not practiced. This underscores the need for RMC not only to promote uptake of facility-based deliveries, but also to improve birth outcomes and reduce complications.

Considering that the sexual reproductive health and rights (SRHR) of Ghanaian adolescents are constitutionally entrenched [40], backed by research-based policy [41] and heavily invested in by the government of Ghana and other development partners, improving the quality of maternity care for adolescents should be a national priority. Moreover, whilst studies have strongly linked mistreatment to poor outcomes, there is relatively little insight on specific interventions that successfully promote RMC for adolescents. Against this background of limited information and knowledge about adolescents’ experiences and outcomes with mistreatment during childbirth, this study seeks to characterize the experiences and outcomes of adolescents who face mistreatment during facility-based births. The study seeks to test the outcome of the proposed intervention in promoting RMC during facility-based deliveries for parturient adolescents. This study also presents an opportunity in addressing some existing gaps in knowledge and information for policy and programming for adolescent maternal health and SRHR.

### Study aim

The overall aim of the study is to determine the effectiveness of a training intervention in promoting respectful maternity care for adolescents during facility-based delivery.

### Specific objectives

The specific objectives of this study are to.

1. Synthesise the available evidence relating to the mistreatment and the provision of respectful maternity care for adolescents during facility-based childbirth using a systematic review approach.
2. Estimate the prevalence of mistreatment during facility-based childbirth among adolescents.
3. Identify the risk factors and outcomes of intra-partum mistreatment during facility-based childbirth among adolescents.
4. Document and characterize the experiences of adolescents who suffer mistreatment during facility-based childbirth.
5. Determine the most and least common forms of Respectful Maternity Care offered to adolescent parturients.
6. Determine the effectiveness of an intervention in promoting respectful maternity care and increasing satisfaction with facility-based births and skilled birth attendance for parturient adolescents.

### Conceptual framework

**Fig. 1:**
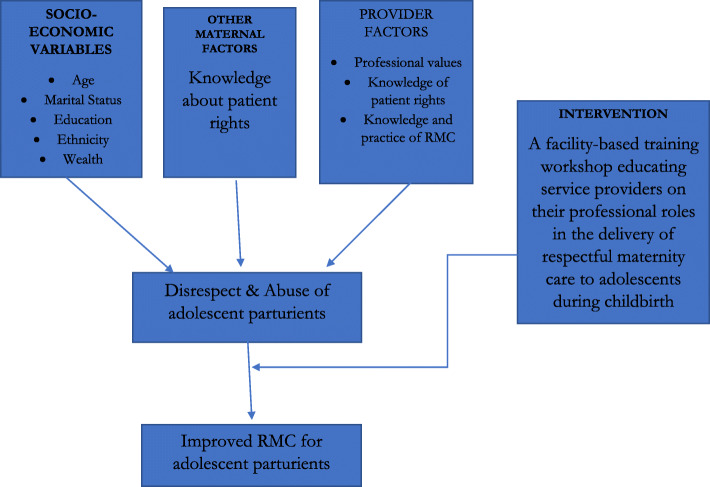
Conceptual Framework

The proposed study is diagrammatically represented in Fig. 1. The conceptual framework is theoretically based and developed from the review of existent literature on intrapartum mistreatment and RMC.

## Methods

### Study settings

The study intervention will be sited in three regions in Ghana: Eastern, Central and Greater-Accra regions. The intervention sites are public health facilities in the Lower Manya Krobo, Upper Manya Krobo district, and the Awutu-Senya East Municipality. The control sites will be in the Ada East, Shai-Osudoku and Effutu Districts. These districts have an average of 11% of their population being adolescent females [42] and reported adolescent pregnancy rates higher than the national average of 14% [22] . The intervention and control sites are selected health facilities which are matched on the type of facilities being district health centres and district hospitals. For each of the intervention sites, a senior member of the district quality of care team will be included as a resource person in the RMC trainings.

### Study design

The study is a non-equivalence pre-test post-test quasi-experimental study. Both qualitative and quantitative methods will be used in data collection.

### Quantitative study population

Study participants in the quantitative phase will be adolescent females between the ages of 10 and 19 years old who deliver at the participating facilities.

### Qualitative study population

The first group of participants in the qualitative phase of this study are adolescent females between the ages of 10 and 19 years old who deliver at the participating study sites. The second group of participants are health providers in full time employment at the study sites. Adolescents and health professionals alike will be invited to participate in the study based on the study inclusion criteria and will be enrolled based on their consent to participate. Adolescents will be invited to participate in In-Depth Interviews (IDIs) and the professionals will participate in Key Informant Interviews (KIIs).

### Inclusion criteria

Adolescents will be deemed eligible for participation if they satisfy the following criteria.

i. Between the ages of 10 and 19 years.
ii. Present at any of the selected health facilities for delivery, antenatal care within their third trimester or post-natal care within two weeks of delivery.
iii. Consent to participate in the study.

Eligibility criteria for health professionals are.

i. Health providers including midwives and administrators who are in full time employment at the selected health facilities.
ii. Consent to participate in the study.

### Exclusion criteria

Health professionals and adolescents will be excluded based on the following criteria

i. Decline to participate
ii. Are physically or mentally ill or indisposed
iii. Did not deliver at the facilities in the study if they are adolescents or are not obstetric care providers or administrative staff of the facilities

### Sample size determination

The minimum sample size for adolescents is obtained observing the assumptions that the estimated proportion of women who encounter mistreatment during childbirth is 18% [43] and the proposed intervention may reduce this by 10 percentage points. Additionally, a 5% margin of error, a 95% significance level and 80% power are estimated.

- N = estimated sample size for one arm.
- Za = Critical value at confidence interval of 95% (1.96).
- Zb = Critical value at power of 80% (0.842).
- d = Actual difference in proportion between control and intervention groups (0.1).
- P = Average of difference in proportion of both groups

$$ \mathrm{N}=2\times \frac{{\left( Z\alpha + Z\beta \right)}^2}{d^2}\times p\left(1-p\right) $$

An intra-cluster design effect of 1.11 and a non-response adjustment of 10% is factored to obtain a minimum sample size of 392.

A minimum of 10 healthcare participants and 10 adolescents will be included in qualitative Key Informant Interviews It is anticipated that the number of interviews will allow a sample size large enough to allow data saturation to understand the phenomenon of mistreatment from both provider and client perspectives.

### Sampling procedure

Multistage, stratified and systematic sampling will be used to recruit study participants from the facilities where they attend. Using data available from the 2010 Population and Housing Census, three out of the formerly ten regions in Ghana with adolescent pregnancy rates higher than the national average (14%) were randomly selected. Subsequently, six districts were purposefully selected from these regions considering appropriateness for the intervention, available resources, and geographical non-proximity between intervention and control facilities. Facilities in the intervention and control arms of the study were matched based on the number of adolescent pregnancies delivered annually using the Ghana Health Service District Health Information Management System (GHS-DHIMS) data from 2018 as a guide.

All eligible adolescent participants who attend the participating intervention and control facilities for antenatal care in their third trimester and delivery will be invited to enrol in the study by a trained research assistant. The nature of the study and all ethical concerns will be explained to the candidate prior to their enrolment. Enrolment will be run on a cumulative bases until the minimum sample size is satisfied. Some adolescents will be purposively selected to participate in the adolescent IDIs based on their characteristics which is aimed at achieving maximum variability of participants. Each district will have a health professional who works in administrative capacity and in a caregiving capacity invited to participate in the Key Informant Interviews.

### Measurement tools

Quantitative data will be collected with the aid of an electronically-based structured questionnaire adapted from Bohren and colleagues’ study [44] which has been modified to suit the local context. The tool is designed to collect data on the socio-demographic characteristics of adolescents and their knowledge on health and sexual reproductive rights. The tool will additionally collect data on the labour experience including the provider-patient encounter and relationships, encounters of mistreatment and RMC, and health facility factors.

Qualitative data collection will be done with the aid of interview guides adapted from the studies by Bohren et al. [44] and Afulani and colleagues [45]. The tool is specifically developed to collect data on the varying forms of mistreatment within the local context. Sections of the guide probe for an in-depth discussion on the themes of knowledge on health and sexual reproductive rights, provider-patient interactions, encounters of mistreatment and RMC and health facility factors. For professionals, the interview will probe for the role of healthcare providers in the labour experience especially in the administration of RMC.

### Data collection procedure

The quantitative interviews will be administered by trained research assistants. Interviews will be administered both at baseline and endline (from day 1 following the intervention). They are estimated to last between 30 and 45 min per participant and will be held at the home of the participant or a convenient private venue outside the health facility within two weeks after delivery. This venue arrangement is considered important to create an enabling environment which allows free and candid dialogue with the participant which may not be possible at the health facility due to fear of retaliation from providers. This time interval between delivery and the interview is also considered adequate for the parturient to recuperate without introducing significant recall bias. Interviews with adolescent participants will be in the most commonly spoken languages in the district which are English, Twi, Dangme or Krobo, based on the preference of the participant. This will allow the participants to express themselves freely in the language they are most comfortable with.

Qualitative IDIs will be conducted with selected adolescent participants and KIIs with health professionals. Interviews will be audio-taped, with prior permission from the participant. Additionally, notes will be taken to capture non-verbal nuances and expressions which may aid in better analysis and interpretation of the audio information. The number of interviews will continue until a saturation of information is reached. Each interview is estimated to last between 45 and 60 min. For adolescents, IDIs will take place at a location convenient to the participant outside the facility within two weeks after delivery. Similar to the quantitative interviews, the qualitative interviews will be conducted within a post-recovery timeframe which mitigates the probability of recall bias. Based on the preference of the participant, IDIs will be conducted in English, Twi, Ga-Dangme or Krobo as done in the quantitative interviews. The KIIs will be conducted within the facilities where the staff work and will be conducted in English.

### Data quality assurance activities

The quality of the study and data collected will be assured via the following means

1. Intervention-Control Allocation: The intervention and control facilities are selected based on the similarity of their characteristics. This includes the type of facility, proportion of adolescent deliveries per year and the socio-demographic characteristics of the community. The geographic non-proximity of the facilities was also highly considered to avoid contamination of the control communities.
2. Research Assistants Recruitment, Training & Monitoring: Research assistants will be selected based on the following criteria
  - Sex: female
  - Age: 18 to 25 years
  - Academic qualification: minimum of a secondary school certificate
  - Technical proficiency: smartphone literacy
  - Language proficiency: at least one of the languages (Ga-Dangme/ Krobo/Twi)
  The age group and sex of the assistants will help establish rapport between the assistants and participants. This will also help minimize response desirability bias on the side of participants. The educational and technical criterion are necessary to ensure that the assistants understand and effectively carry out their roles in the study to collect quality data. The assistants will undergo a two-day training on the theoretical background of the study, enrolment and obtaining consent from participants, administration of questionnaires and facilitation of interviews, as well as ethical issues. The principal investigator will always closely supervise the research assistants to ensure their observance of study protocols and ethical codes
3. Data Collection Instruments: The data collection instruments are adapted from a previous multi-country study (which included Ghana) which included parturient women of all ages [46] instruments will be adapted for use with adolescents. The tools will be pretested on 5% of the sample size in a pilot facility in the Kpone-Katamanso district and modified accordingly based on findings from the pilot. The pilot district was selected based on its similarity to study sites in having an adolescent birth rate of greater than 11%.
4. Data Collection Timing and Location: Data collection at baseline will be over a 3-month period. Endline data collection will start from day 1 after the intervention until three-months post-intervention. The venue and time interval between delivery and the adolescent surveys will allow participants express themselves freely without fear of victimization by health providers. This will also allow the participants enough recovery time from childbirth with minimal recall bias.
5. Data Monitoring: Data collection will be conducted in real time electronically. The data will be monitored daily to allow early detection of discrepancies and resultant remedial action. Transcripts from audio interviews will be reviewed by an independent listener who is fluent in the language and did not conduct or transcribe the interview to audit for accuracy and completeness.

### Data processing and statistical analysis

Both baseline and endline data will be reviewed and edited for completeness and consistency daily. The primary outcome variable of interest is defined as disrespectful intrapartum care which is measured as a response of “Yes” to any of the nine mistreatment questions. The explanatory variables of interest include socio-economic status, educational level, marital status and knowledge of health rights. Data will be collected on the KoBoCollect platform, synced onto a web server and downloaded in Ms-Excel format to the principal investigator’s computer. The data will be exported in STATA version 16 for cleaning and analyses. Descriptive statistics as well as binary and multiple logistic regressions will be computed. Additionally, Difference in Differences (DID) will be calculated to determine the net RMC training intervention effect on mistreatment and RMC. Finally, statistical significance of the RMC training intervention effect will be evaluated using adjusted prevalence ratio, adjusted odds/risk ratio at 95% confidence interval (CI) and *P*-Value of less than 0.05.

Audiotapes from qualitative interviews will be translated and transcribed verbatim into Ms-Word format in English. Themes will be identified and coded in Ms-Excel and exported into NVivo for analyses. Quotes will be narrated and cited in participant codes.

Data analysis for qualitative and quantitative data will be conducted separately. Subsequently, inferences from both qualitative and quantitative results will be jointly used to draw conclusions.

### Intervention

The proposed intervention aims to educate and inform midwives about the concepts of mistreatment and RMC. The training additionally seeks to help participants explore and shift their current values and beliefs about mistreatment of adolescent parturients towards more positive behaviours. It also aims to encourage the practice of RMC by promoting adherence to positive professional codes of conduct, ethics, roles, values and attitudes. The proposed intervention is a training module facilitated with the aid of a resource package adapted from the White Ribbon Alliance/ USAID “Respectful Maternity Care for Healthcare Workers: Tackling Disrespect & Abuse During Facility-Based Childbirth” project [47]. Additionally, the structure and content of the module is influenced by evidence from previous studies and systematic reviews on mistreatment and RMC [48–54]. This course will be presented in the format of presentations, case studies, role plays, discussions and evaluations. The content will cover the adolescent and their reproductive health rights, the Ghana Health Service (GHS) code of professional ethics, Nurses and Midwives Code of Conduct, definitions and typologies of mistreatment and RMC as well as their outcomes to the woman, neonate, professional and health system. The content will also cover the professionals’ role in the delivery of RMC. The training workshops will be implemented by the principal investigator supported by a facilitator who is member of the District Quality of Care team. Each workshop will be a two-day event. The first day will cover a theoretical introduction of the concepts of mistreatment and RMC and the role of the obstetric professional. The second day will be a series of case studies and role plays to apply the theoretical basis from day 1. The facilities will additionally be provided with visible posters and materials as a constant reminder of the contents of the training programme.

There will be no intervention conducted in the control facilities.

### Expected outcomes

It is anticipated that at the end of this proposed study, the following outcomes will be achieved per each objective as summarised in Table 1.

**Table 1:** Expected Outcomes of the Study

Specific Objective 1: Synthesise the available evidence relating to the mistreatment and the provision of respectful maternity care for adolescents during childbirth using a systematic review approach	Conduct a review of the existing published and grey literature on mistreatment and RMC globally for adolescents. Identify the reported prevalence and associated factors of mistreatment Identify interventions that address mistreatment and promote RMC
Specific Objective 2:Estimate the prevalence of mistreatment during childbirth among adolescents	Conduct a baseline assessment of mistreatment across adolescent participants in intervention and control facilities.
Specific Objective 3: Identify the risk factors and outcomes of intra-partum mistreatment among a cross-section of adolescents	Interview health professionals to explore their perceptions on the associated risk factors of mistreatment and its outcomes on adolescents, health professionals and the national health system.
Specific Objective 4: Document and characterize the experiences of a group of adolescents who suffer mistreatment during childbirth	Interview adolescents on their detailed perceptions and personal experiences with intrapartum mistreatment
Specific Objective 5: Determine the most and least common forms of Respectful Maternity Care offered to adolescent parturients	Conduct an evaluation of the most and least commonly occurring RMC practices offered in the selected intervention and control facilities
Specific Objective 6: Determine the effectiveness of an intervention in promoting respectful maternity care and increasing satisfaction with facility-based births and skilled birth attendance for parturient adolescents	Implement an RMC training intervention across the selected intervention communities Conduct an endline assessment across intervention and control facilities to determine the prevalence of mistreatment Compute the differences in mistreatment and RMC between intervention and control facilities at baseline and endline.

## Discussion

This study will firstly establish the prevalence of mistreatment among parturient adolescents. Several studies worldwide have reported the prevalence of mistreatment among women in general [3, 55–58]. This proposed study, however, is among the first of its type to focus exclusively on the mistreatment of adolescents. Furthermore, the study will determine the effectiveness of an intervention in promoting RMC in Ghana and reducing mistreatment. Previous studies have employed interventions that target facility users such as holding maternity open birth days and community workshops to improve public awareness about mistreatment [6, 43]. Mediation and resolution of past incidences of mistreatment whilst preventing future events has also been managed by educating communities on their SRHR whilst providing a means of legal redress [59]. Whilst these interventions may be helpful for older women, they may not be ideal for younger adolescents as they may not possess the autonomy required to properly exercise these facilities. Some interventions have also targeted providers with evidence-based information by providing access to the World Health Organization Reproductive Health Library (RHL) [60] and promoting advocacy for birth companions [61]. A study by Abuya and colleagues in Kenya recorded a 7% reduction in mistreatment following the implementation of a multi-component intervention at the policy, facility and community levels [43]. However, there was no special focus in these studies on adolescent sub-groups and their peculiar experiences or needs. This study therefore seeks to provide insight on interventions that may better address the peculiar maternal care needs of adolescent parturients.

### Limitations

There are a few limitations in this study which measures have been taken to address. Firstly, it was not possible to randomly assign the study sites due to geographical and health system constraints. It is therefore possible that there will be observed and unobserved differences in the baseline measures of the intervention and comparison groups that may account for differences in outcomes. However, the study sites were selected based on their similarities in characteristics such as type of facility and socio-geographic factors. Furthermore, the use of difference-in-differences analytic methods in the analyses of data will allow the account of both observed and unobserved confounders. Another limitation that may be faced is the employment of new staff into intervention facilities after the intervention has been conducted. To mitigate this limitation, we shall take note of new staff and encourage the training module to be shared during their staff orientation.

### Dissemination

Dissemination strategies have been drawn for results emanating from this study. Oral and poster presentations of the results will be made to the participating facilities as well as district and regional directorates of the Ghana Health Service. Furthermore, the results of the specific objectives in this proposal will be published in peer review journals. The data management procedures, study materials including questionnaires and implementation frameworks of the study will be made upon reasonable request to the corresponding author.

## Data Availability

The data management procedures, study materials including questionnaires and implementation frameworks of the study will be made upon reasonable request to the corresponding author.

## Abbreviations

ASRHR: Adolescent Sexual Reproductive Health & Rights
CHPS: Community-based Health Planning & Services
DHIMS: District Health Information Management Systems
FBD: Facility-Based Delivery
GDHS: Ghana Demographic & Health Survey
GHS: Ghana Health Service
GMHS: Ghana Maternal Health Survey
HRP: Human Reproduction Programme
IDI: In-Depth Interview
KII: Key Informant Interview
RMC: Respectful Maternity Care
SBA: Skilled Birth Attendance
SDG: Sustainable Development Goals
SRHR: Sexual Reproductive Health & Rights
WHO: World Health Organization
